# Does antenatal micronutrient supplementation improve children’s cognitive function? Evidence from the follow-up of a double-blind randomised controlled trial in Nepal

**DOI:** 10.1136/bmjgh-2017-000527

**Published:** 2018-02-28

**Authors:** Sophiya Dulal, Frédérique Liégeois, David Osrin, Adam Kuczynski, Dharma S Manandhar, Bhim P Shrestha, Aman Sen, Naomi Saville, Delan Devakumar, Audrey Prost

**Affiliations:** 1 Mother and Infant Research Activities (MIRA), Kathmandu, Nepal; 2 Institute of Child Health, University College London, London, UK; 3 Institute for Global Health, University College London, London, UK; 4 Department of Clinical Neuropsychology, Great Ormond Street Children’s Hospital, London, UK

**Keywords:** child health, nutrition, clinical trial, public health

## Abstract

**Introduction:**

Multiple Micronutrient (MMN) supplementation during pregnancy can decrease the proportion of infants born low birth weight and small for gestational age. Supplementation could also enhance children’s cognitive function by improving access to key nutrients during fetal brain development and increasing birth weight, especially in areas where undernutrition is common. We tested the hypothesis that children whose mothers received MMN supplementation during pregnancy would have higher intelligence in early adolescence compared with those receiving Iron and Folic Acid (IFA) only.

**Methods:**

We followed up children in Nepal, whose mothers took part in a double-blind Randomised Controlled Trial (RCT) that compared the effects on birth weight and gestational duration of antenatal MMN versus IFA supplementation. We assessed children’s Full Scale Intelligence Quotient (FSIQ) using the Universal Non-verbal Intelligence Test (UNIT), and their executive function using the counting Stroop test. The parent trial was registered as ISRCTN88625934.

**Results:**

We identified 813 (76%) of the 1069 children whose mothers took part in the parent trial. We found no differences in FSIQ at 12 years between MMN and IFA groups (absolute difference in means (diff): 1.25, 95% CI −0.57 to 3.06). Similarly, there were no differences in mean UNIT memory (diff: 1.41, 95% CI −0.48 to 3.30), reasoning (diff: 1.17, 95% CI −0.72 to 3.06), symbolic (diff: 0.97, 95% CI −0.67 to 2.60) or non-symbolic quotients (diff: 1.39, 95% CI −0.60 to 3.38).

**Conclusion:**

Our follow-up of a double-blind RCT in Nepal found no evidence of benefit from antenatal MMN compared with IFA for children’s overall intelligence and executive function at 12 years.

**Key questions**

**What is already known about this topic?**

- Three systematic reviews have called for more evidence on the effects of antenatal micronutrient supplementation on children’s cognitive function.
- Five follow-up studies of trials found no effect of supplementation on children’s cognitive function in childhood and early adolescence, one found a small positive effect at 12 months, which subsequently disappeared at 7–10 years, and one found a small positive effect on procedural memory but not general intellectual ability at 12 years.

**What are the new findings?**

- Our follow-up study, the first from a low-income country, found no differences in IQ or executive function at 12 years between children whose mothers received multiple micronutrient supplementation in pregnancy and those who did not.

**Recommendations for policy**

- The evidence from existing trials conducted in low- and middle-income countries suggests that antenatal MMN supplementation leads to null or small effects on children’s long-term cognitive function.

## Introduction

An estimated 250 million children globally are denied the opportunity to reach their full developmental potential due to the combined effects of poverty and undernutrition.^1^ Poor nutrition during pregnancy contributes to maternal morbidity and mortality, increases the risks of low birth weight and poor development, and exacerbates the risk of chronic disease in adulthood.^2 3^ Many pregnant women face micronutrient deficiencies: an estimated 15.3% globally lack vitamin A, 28.5% lack iodine and 38% have iron-deficiency anaemia.^1 4^ Nutritional deprivation in pregnancy can alter neural growth in the fetus and affect cognitive functioning, hindering children’s chances in school and contributing to the intergenerational transmission of poverty.^5–9^

The World Health Organisation (WHO) currently recommends Iron and Folic Acid (IFA) supplementation in pregnancy to improve maternal and perinatal outcomes, but not Multiple Micronutrient (MMN)supplementation.^10–12^ Could antenatal micronutrient supplementation improve children’s long-term cognitive functioning? Current evidence is inconclusive. Supplementation with single micronutrients during pregnancy appears to have little influence on children’s cognitive outcomes: 10 trials conducted in high-income, middle-income and low-income settings found no effects of antenatal iron, zinc, vitamin A or choline supplementation on cognitive functions.^13–20^ Supplementation with MMN, on the other hand, could affect children’s cognitive functions via three related pathways. The first is via the direct effect of gestational nutrition on brain development: iodine, iron, zinc, copper, folic acid and vitamin B_12_ play important roles during brain growth, a significant portion of which occurs between 34 weeks of gestation and 2 years.^6 21^ A second pathway is via epigenetic changes: micronutrient availability influences the programming of later development via effects on genes linked to neural growth and immunity.^22^ Finally, antenatal MMN supplement could improve cognitive functioning via changes in birth outcomes, including birth weight, though it is unclear whether increased birth weight leads to improved cognitive development or whether both are joint consequences of a nourishing uterine environment.^23 24^

Three recent systematic reviews have called for further follow-up studies of the effects of MMN supplementation on cognitive outcomes.^23 25 26^

Our study aimed to follow up children whose mothers took part in a trial of antenatal supplementation conducted in Nepal between 2002 and 2004.^27^ The trial tested the effect of the United Nations International Multiple Micronutrient Preparation (UNIMMAP), which contains 15 vitamins and minerals, versus IFA only.^28^ Between August 2015 and March 2016, we followed up children born during the trial to determine whether those exposed to antenatal MMN supplementation had a higher IQ at 12 years compared with those exposed to IFA only.

## Methods

### Setting

The follow-up study was conducted in Dhanusha district, in the lowland Terai region of Nepal. Dhanusha has around 768 000 inhabitants; most households are rural and over half are involved in agriculture.^29^ Maternal and child undernutrition are common in the Terai : 23% of women aged 15–49 have a Body Mass Index (BMI) below 18.5 kg/m^2^, almost half (52%) of pregnant women are anaemic, and 36.7% of children under 5 are stunted.^30^ Opportunities for formal learning and stimulation during early childhood are limited: around half (49%) of children aged 36–49 months attend a preschool programme and 5% of children under 5 have access to children’s books.^29 31^

### Design and participants

We followed up children born during the Randomised Controlled Trial (RCT) of antenatal micronutrient supplementation conducted in Janakpur, the district headquarters, between 2002 and 2004.^27^ The trial tested the effect supplement, the UNIMMAP, taken daily from the 12th week of gestation—at minimum—until delivery, compared with a daily supplement of iron (60 mg) and folic acid (400 µg) recommended by the government. The UNIMMAP contains vitamin A 800 µg, vitamin E 10 mg, vitamin D_5_ µg, vitamin B_1_ 1.4 mg, vitamin B_2_ 1.4 mg, niacin 18 mg, vitamin B_6_ 1.9 mg, vitamin B_12_ 2.6 µg, folic acid 400 µg, vitamin C 70 mg, iron 30 mg, zinc 15 mg, copper 2 mg, selenium 65 µg and iodine 150 µg. The trial’s primary outcomes were birth weight and gestational duration. A total of 1200 pregnant women visiting the antenatal clinic of Janakpur Zonal hospital were randomly allocated to the intervention or control arm. Supplements in both arms looked, smelled and tasted identical. Women enrolled in the trial were followed up every 2 weeks through home and clinic visits, during which the study team assessed their adherence to supplements. The exclusion criteria were gestation beyond 20 weeks, multiple pregnancies, fetal abnormality detected by ultrasound and existing maternal illness that might compromise the pregnancy outcome. The trial team recorded 1069 deliveries and infants were followed up at birth and after 1 month. Women, their family members and the research team were masked to allocation.

Its primary outcomes were birth weight and gestational duration. A total of 1200 pregnant women who visited an antenatal clinic in Janakpur Zonal hospital were randomly allocated to the intervention or control arm, and followed up every 2  weeks through home and clinic visits. The trial team recorded 1069 deliveries, and infants were followed up at birth and after 1  month. Women, their family members and the research team were masked to allocation. Antenatal exposure to the UNIMMAP led to an increase of 77 g (95%  CI 24 to 130) in mean birth weight compared with IFA.^27 32 33^ Gains in weight were still present at 2.5 years, but disappeared by 8.5 years. 27 32 33

### Procedures

We sought to locate children born during the parent trial and identified during the most recent follow-up in 2012.^27 33^ A member of the study team visited each family to obtain informed consent, interviewed mother and child, and invited them for a cognitive assessment. We made at least three attempts to invite and assess each child. We excluded children who were ill on all three attempts or had severe hearing, visual or motor impairments. To find and assess children who had migrated, we visited the nearby districts of Sarlahi and Siraha, as well as Kathmandu and Makwanpur district, where some children had been found previously.

### Outcome measures

Our main outcome of interest was the Full Scale Intelligence Quotient (FSIQ) measured using the Universal Non-verbal Intelligence Test (UNIT).^34^ The UNIT was developed to test children irrespective of country of origin, sex or language. It includes subtests of symbolic memory, cube design, spatial memory, analogical reasoning, object memory and the ability to work through mazes. It is suitable for children aged 5–17 years, relies on non-verbal (gestural) instructions and feedback, takes around 45 min to administer and has been used previously in Nepal.^35^ The UNIT allows calculation of the FSIQ, a Memory Quotient (MQ), a Reasoning Quotient (RQ), which captures the ability to solve problems using information, a Symbolic Quotient (SQ), which assesses the ability to understand and process language, and a Non-symbolic Quotient (NSQ), reflecting the ability to perceive, recognise, sequence, organise and integrate information.^34^

We used a counting Stroop test to assess children’s executive function.^36^ The number–quantity Stroop task involved three conditions.^37 38^ In each condition, the children were asked to name the quantity of items in rows of one to seven identical items. In the baseline condition, children were asked to name the quantity of X in a row (eg, the correct response to XX is ‘two’). Second, in the congruent condition, they were asked to name the quantity of digits in a number with the equivalent digits (eg, 55555=‘five’). Finally, in the third, incongruent condition, they were asked to name the quantity of digits in a number with the non-equivalent digits (eg, 1111=‘four’). We recorded the time that children took to correctly name all items for each condition. We then derived two scores from these totals. An *interference score* was calculated by subtracting the baseline from the incongruent condition. This represented the potentially disruptive effect of automatic reading of the incongruous digits on quantity naming. A *facilitation score* was calculated by subtracting results for the congruent condition from those for the baseline, representing the enhancing effect of congruent digits.

Training of the testers was comparable to that for the US standardisation of the UNIT. The principal investigator (SD) was trained by two experienced UK-based psychologists (one clinical, one experimental, AK and FL) in the general principles of psychometric assessment and procedures specific to the UNIT and the counting Stroop test. She recruited two testers from Dhanusha district who were fluent in the local language (Maithili) and had a background in education and social science. The testers were trained for 15 days to administer the tests and practised under supervision for a week with children aged 10–13. We video-recorded the testers’ practice administrations of the UNIT. These were checked by the two experienced psychologists to ensure adherence to standardised testing procedures.

SD and the two other testers administered the UNIT and Stroop tests in an office with minimal distractions located in Janakpur. The testers recorded the children’s UNIT raw and scaled scores on a form with a unique child identification number. We checked each form individually and confirmed the scores using the proprietary CompuScore (V.1.1) UNIT software. These audited raw, scaled and standardised scores were double-entered into a Microsoft Access database. We identified discrepancies between first and second entries and resolved them by consulting the original forms.

To check the quality of UNIT assessments, we video-recorded 16 randomly selected assessments. Review of the videos by two experienced psychologists indicated that the cognitive testing procedures adhered closely to the standardised administration during data collection.

**Figure 1:**
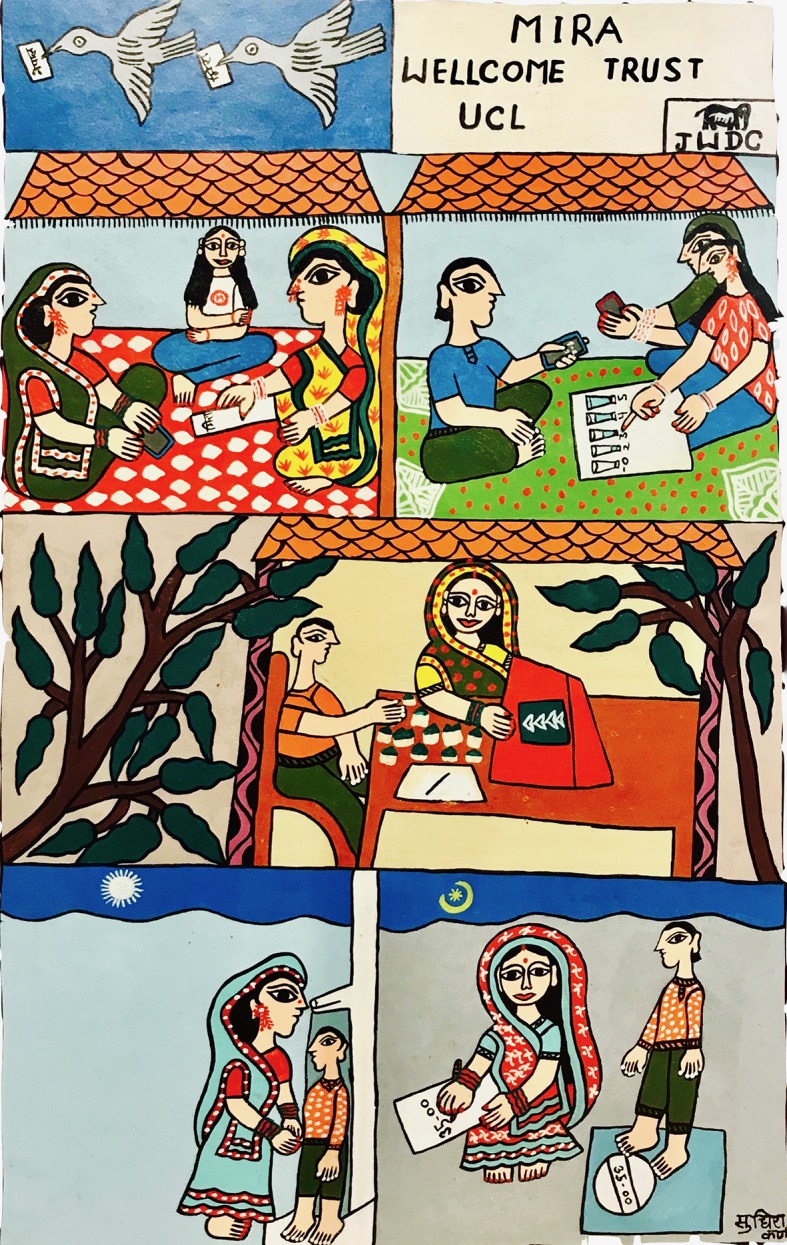
Mithila painting depicting consent taking, interviewing, and a child participating in the Universal Non-verbal Intelligence Test, Stroop test and anthropometry.

In addition to intelligence and executive function, we collected data on factors that could confound the effects of MMN supplementation on IQ, including the quality of the home environment, the child’s and parents’ educational attainment, the mother’s mental health and the child’s current nutritional and health status (height, weight, dietary recall, morbidity recall and mental health). We used a Shorr board, accurate to 1 mm, to measure children’s height and a Tanita HS302 solar scale, accurate to 100 g, to measure weight. We assessed mothers’ mental health with the 12-item General Health Questionnaire, which had been used previously in this setting.^39^ We used the Depression Self-Rating Scale (DSRS) and Screen for Child Related Anxiety Disorder (SCARED) tools to assess children’s mental health. The DSRS has been adapted and validated for use in Nepal.^40^ Finally, we used the Early Adolescent Home Observation Tool (HOME) to assess the quality of the children’s home environment. We followed the Home Inventory Administration Manual’s observation techniques and structured interview format.^41^ We removed one out of the 60 items in HOME because it was culturally sensitive: item 36 asked whether a parent had provided guidance to the adolescent about responsible sexuality and physical hygiene in the past year. In Dhanusha, parents do not commonly talk about sexuality with their children, sowe removed the question to avoid discomfort during the interview process. The interview format was translated into Nepali and Maithili, pretested and adapted before data collection. Questionnaire information was collected with smartphones using Open Data Kit.^42^ Interviewers received a month of training to ensure that they had the same understanding of tools and data collection processes. We piloted the tools in the community. Data collection was supervised and monitored throughout the follow-up period. Data were downloaded weekly and checked by SD. The data collection team and analyst (SD) were blind to the allocation of children in the parent trial. Figure 1 is a Mithila painting depicting the study team requesting consent from parents, interviewing a mother and child, and a child participating in the Stroop, UNIT and anthropometry.

### Statistical methods

As the sample size was fixed by that of the parent trial, we estimated the smallest differences in mean UNIT scores that our study could detect with 80%–90% power, and an alpha of 0.05. Even with 16% attrition from the last follow-up (from 841 to 700 children), the study would have 87.1% power to detect a small (3.5) difference in mean IQ scores between groups assuming a SD of 15, or an effect size of 0.23 using Cohen’s *d*.

Primary analyses followed intention-to-treat principles. We compared the characteristics of participants identified and those lost to follow-up by trial allocation using descriptive statistics. We used WHO Child Growth Standards for children to generate Z scores for weight, height and BMI-for-age.^43^ We used independent two-sample t-tests to examine differences between intervention and control groups in means of standardised scores for the MQ, RQ, SQ, NSQ and FSIQ. We used linear regression models with and without adjustment for covariates. We examined the associations between each of the covariates in tables 2 and 3 with FSIQ scores and with one another through univariable analyses. We then selected covariates that were associated with FSIQ and were not collinear by visually examining their associations in a correlation matrix. A priori, and building on previous studies, we decided to carry out subgroup analyses to assess the effect of supplements on children’s FSIQ by adherence, maternal BMI, haemoglobin level in pregnancy and birth weight. Because the parent trial had detected a small differential effect of MMN on birth weight by sex, we also examined the association between exposure to the intervention and FSIQ by sex. Statistical analyses were conducted using STATA (V.13.1).

**Figure 2:**
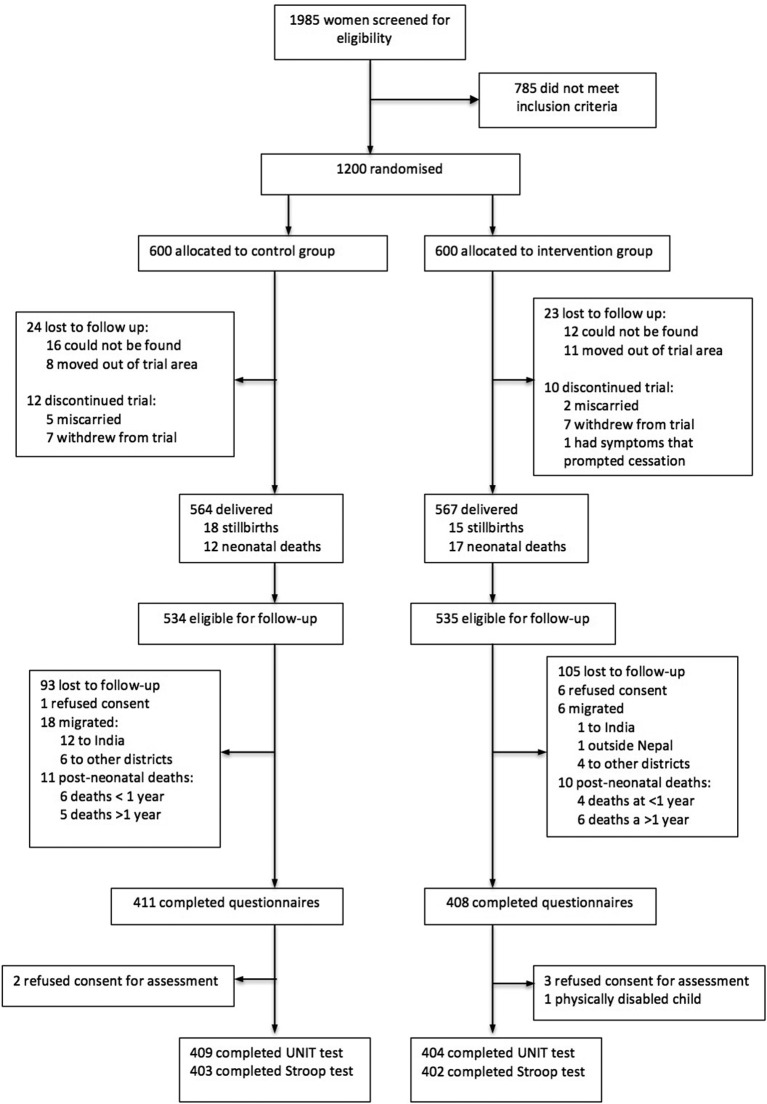
Study profile. UNIT, Universal Non-verbal Intelligence Test.

**Table 1:** Characteristics of children retained at 12 years and those lost to follow-up

	12-year follow-up		Lost to follow-up	
	Control	Intervention	Before end of trial	After end of trial
	n (%)	n (%)	n (%)	n (%)
Location				
Urban	194 (47%)	190 (47%)	47 (68%)	196 (62%)
Rural	215 (53%)	214 (53%)	22 (32%)	122 (38%)
District				
Dhanusha	339 (83%)	325 (80%)	59 (86%)	265 (83 %)
Mahottari	70 (17%)	75 (19%)	10 (14%)	51 (16%)
Sarlahi	0	2 (0.5%)	0	1 (0.3%)
Siraha	0	2 (0.5%)	0	1 (0.3%)
Main household livelihood				
No work	47 (11%)	46 (11%)	1 (1 %)	36 (11%)
Farming	70 (17%)	65 (16%)	7 (10%)	39 (12%)
Salaried	150 (37%)	167 (41%)	34 (49%)	162 (51%)
Small business	80 (20%)	75 (19%)	19 (28%)	49 (15%)
Waged labour	50 (12%)	43 (11%)	5 (7%)	21 (7%)
Student	7 (2%)	4 (1%)	3 (4 %)	3 (1 %)
Out of country	5 (1%)	4 (1%)	0	8 (3%)
Mother’s age at enrolment				
<20 years	118 (29%)	124 (31%)	20 (29%)	99 (31%)
20–29 years	269 (66%)	266 (66%)	44 (64%)	206 (65%)
≥30 years	22 (5%)	14 (3%)	5 (7%)	13 (4%)
Ethnic origin, N				316
Dalit Plains	9 (2%)	12 (3%)	2 (3%)	6 (2%)
Muslim	21 (5%)	29 (7%)	8 (12%)	19 (6%)
Janjati Hills	7 (2%)	9 (2%)	2 (3%)	14 (4%)
Other Terai (Plains) groups	289 (70%)	266 (66%)	38 (55%)	193 (61%)
Brahmin Chhetri Hills	23 (6%)	21 (5%)	6 (9%)	22 (7%)
Brahmin Chhetri Plains	60 (15%)	67 (17%)	13 (19%)	62 (20%)
Land ownership				
No land	19 (5%)	23 (6%)	6 (9%)	20 (6%)
<30 dhur (about 500 m^2^)	281 (69%)	280 (69%)	45 (65%)	219 (69%)
≥30 dhur	109 (27%)	101 (25%)	18 (26%)	79 (25%)
Appliance score, N			68	
Motor vehicle, TV or refrigerator	209 (51%)	200 (50%)	36 (53%)	168 (53%)
Sewing machine, cassette player, camera, fan, bullock cart, wall clock, radio, iron or bicycle	145 (35%)	139 (34%)	21 (31%)	96 (30%)
None of the above	55 (13%)	65 (16%)	11 (16%)	54 (17%)
Maternal education at enrolment				
None	199 (49%)	200 (50%)	27 (39%)	118 (37%)
Primary	37 (9%)	31 (8%)	16 (23%)	39 (12%)
Secondary or higher	173 (42%)	173 (43%)	26 (38%)	161 (51%)
Mother’s body mass index at enrolment, N	408	403		
<18.5 kg/m^2^	126 (31%)	120 (30%)	23 (34%)	73 (23%)
≥18.5 kg/m^2^	282 (69%)	283 (70%)	45 (66%)	245 (77%)
Parity at birth of index child				
0	179 (44%)	175 (43%)	33 (48%)	153 (48%)
1–2	176 (43%)	196 (49%)	28 (40%)	137 (43%)
≥3	54 (13%)	33 (8%)	8 (12%)	28 (9%)
Preterm, N			8	
(<37 weeks’ gestation by ultrasound assessment)	25 (6%)	25 (6%)	3 (38%)	48 (15%)
Place of birth, N			5	
Hospital	212 (52%)	246 (60%)	–	188 (59%)
Home	187 (46%)	156 (39%)	3 (60%)	116 (36%)
On the way	10 (2%)	2 (1%)	2 (40%)	14 (4%)
Child sex, N	407			
Girl	203 (50%)	196 (49%)	–	160 (51%)
Boy	204 (50%)	208 (51%)	–	152 (49%)
Total	409 (100)	404 (100)	69 (100)	318 (100)

**Table 2:** Characteristics of parents, the home environment and children at follow-up, by trial allocation status

	Control, no (%)	Intervention, no (%)
Child’s primary caregiver		
Mother	383 (94%)	384 (95%)
Father	10 (2%)	4 (1%)
Other	16 (4%)	16 (4%)
Mother’s education		
None	184 (45%)	183 (45%)
Pre-primary	7 (1%)	1 (0.3%)
Primary	39 (10%)	47 (12%)
Secondary or higher	176 (43%)	171 (42%)
Studied Urdu, Sanskrit or non-formal education	3 (1%)	2 (0.5%)
Mother’s literacy		
Can read easily or with some difficulty	217 (53%)	214 (53%)
Cannot read	191 (47%)	190 (47%)
Mother’s psychological distress (GHQ-12)		
No ormild distress (<6)	346 (85%)	343 (85%)
Distress (≥6)	63 (15%)	61 (15%)
Father’s education		
None	84 (21%)	80 (20%)
Pre-primary	–	1 (0.3%)
Primary	38 (9%)	36 (9%)
Secondary or higher	287 (70%)	285 (71%)
Studied Urdu, Sanskrit or non-formal education	–	2 (0.5%)
Child sex and age		
Female	205 (50%)	194 (48%)
Male	204 (50%)	210 (52%)
Mean (SD) age (years)	12.1 (0.4)	12.2 (0.4)
Child schooling		
No schooling	13 (3 %)	6 (1%)
Up to pre-primary level	6 (2%)	4 (1%)
Up to primary level	246 (60%)	238 (59%)
Up to lower secondary level	144 (35%)	155 (38%)
Up to higher secondary level	0	1 (0.3%)
Child’s schooling, N	344	335
Mean no of years (SD)	6.5 (1.6)	6.6 (1.5)
No of siblings		
0	12 (3 %)	13 (3 %)
1–2	259 (63 %)	279 (69%)
≥3	138 (34%)	112 (28%)
Screen child anxiety-related disorder		
No symptoms of anxiety (<3)	203 (50%)	209 (52%)
Symptoms of anxiety (≥3)	206 (50%)	195 (48%)
Depression Self-Rating Scale		
No symptoms of major depression (<14)	364 (89%)	365 (90%)
Symptoms of major depression (≥14)	45 (11%)	39 (10%)
Major illness in the past 12 months		
Yes	33 (8%)	38 (9%)
No	376 (92%)	366 (91%)
Morbidity in the past 7 days		
Fever	40 (10%)	42 (10%)
Diarrhoea	13 (3%)	15 (4%)
Blood in stool	5 (1%)	6 (1%)
Pneumonia	1 (0.2%)	5 (1%)
Fast breathing	2 (0.4%)	7 (2%)
Chest indrawing	3 (1%)	8 (2%)
Cough	72 (18%)	78 (19%)
Total	409 (100)	404 (100)

GHQ-12, 12-item General Health Questionnaire.

**Table 3:** Children’s psychometric test results, by trial allocation

	Control (n=409) No (%)	Intervention (n=404) No (%)	Difference in means Intervention control (95% CI)	P value*	Coefficient (95% CI)†	P value
Standardised UNIT scores						
Full-scale IQ, mean (SD)	76.4 (14)	77.6 (12.3)	1.25 (−0.57 to 3.06)	0.18	1.12 (−0.51 to 2.75)	0.17
Memory quotient, mean (SD)	78.3 (14.6)	79.7 (12.7)	1.41 (−0.48 to 3.30)	0.14	1.23 (−0.50 to 2.95)	0.16
Reasoning quotient, mean (SD)	79.2 (14.3)	80.4 (13.1)	1.17 (−0.72 to 3.06)	0.22	1.15 (−0.61 to 2.92)	0.20
Symbolic quotient, mean (SD)	72 (12·2)	73 (11.5)	0.97 (−0.67 to 2.60)	0.25	0.70 (−0.82 to 2.21)	0.37
Non-symbolic quotient, mean (SD)	86.1 (15.5)	87.5 (13.3)	1.39 (−0.60 to 3.38)	0.17	1.43 (−0.39 to 3.24)	0.12
Counting Stroop test						
Completed Stroop test, N	403	402	–		–	–
Interference score, mean (SD)	1.7 (6.2)	2.3 (6)	0.57 (−0.26 to 1.41)	0.18	0.54 (−0.34 to 1.43)	0.23
Facilitation score, mean (SD)	7.2 (6.2)	6.9 (6)	−0.28 (−1.11 to 0.55)	0.51	−0.28 (−1.17 to 0.60)	0.52

*Unadjusted, derived from independent-samples t-test.

†Adjusted coefficients derived from linear regression models that included residence (rural vs rural), maternal literacy (cannot read vs can read easily or with difficulty), HOME score (continuous), tester (categorical) and children’s age in days as covariates.

HOME, Early Adolescent Home Observation Tool; UNIT, Universal Non-verbal Intelligence Test.

## Results

Between August 2015 and March 2016, we found and assessed 813 (76%) of the 1069 children born during the parent trial: 198 (18%) children could not be located, 7 (1%) refused consent, 24 (2%) had migrated to India or other districts of Nepal, 21 children had died (2%), 5 refused consent and 1 had a disability that prevented them from being assessed. In the most recent follow-up in 2012, 841 (78.6%) of the 1069 children were found, 28 (3.3%) of whom were lost to follow-up in our study. We retained 409/534 (76.6%) of children in the intervention group and 404/535 (75.5%) in the control group. Figure 2 shows the study profile.

Table 1 describes the sociodemographic characteristics of children retained in this follow-up and those lost to follow-up. Participants were similar in most characteristics, though children lost to follow-up were more likely to live in urban areas and have more educated mothers.

Table 2 describes characteristics of the children, their parents and the home environment at the 12-year follow-up. These were well balanced between intervention and control groups. The mean age at follow-up was 12.2 (SD 0.4) in the intervention group and 12.1 years (SD 0.4) in the control group. There were 194 girls (48%) and 210 boys (52%) in the intervention group, compared with 205 girls (50%) and 204 boys (50%) in the control group. Children’s mean number of years of schooling was 6.6 (SD 1.5) in the intervention group and 6.5 (SD 1.6) in the control group. Maternal education, paternal education, HOME scores, current morbidity and symptoms of mental health disorders were similar between groups. The mean HOME scores were 33.8 (SD 7.3) in the intervention group and 33.7 (SD 7.4) in the control group. Scores for symptoms of anxiety as assessed by the SCARED scale were similar between groups (intervention mean: 2.4, SD 1.2; control mean: 2.5, SD 1.3), as were scores for symptoms of major depression (intervention mean: 9.4, SD 2.9; control mean: 9.8, SD 3). As in the 2012 follow-up, there were no differences in children’s anthropometry between groups, either in weight (intervention mean: 31.8, SD 6.9; control mean: 31.5, SD 6.1), BMI-for-age Z scores (intervention mean: −1.3, SD 1.2; control mean: −1.4, SD 1.1), height (intervention mean: 141.2, SD 7.8; control mean 141.5, SD 7.2) or height-for-age Z scores (intervention mean: −1.4, SD 1.1; control mean: −1.4, SD 1).

Standardised UNIT scores were normally distributed in both groups. We found no differences in IQ between children whose mothers had received antenatal MMN supplementation and those who had received IFA only: the mean standardised FSIQ score was 77.6 (SD 12.3) in the intervention group and 76.4 (SD 14) in the control group (absolute difference in means, intervention−control (diff: 1.25, 95% CI −0.57 to 3.06; P=0.17). Similarly, there were no differences in mean UNIT memory (diff: 1.41, 95% CI −0.48 to 3.30; P=0.14), reasoning (diff: 1.17, 95% CI −0.72 to 3.06; P=0.22), symbolic (diff: 0.97, 95% CI −0.67 to 2.60; P=0.25) or non-symbolic quotients (diff: 1.39, 95% CI −0.60 to 3.38; P=0.17). We found no differences in executive function between groups, with similar interference (diff: 0.57, 95% CI −0.27 to 1.41; P=0.18) and facilitation scores (diff: 0.28, 95% CI −1.12 to 0.55; P=0.50) in the Stroop test.

We selected residence, maternal literacy, HOME scores and children’s age as covariates because they were independently associated with FSIQ and not collinear with one another. We observed differences in the mean and distribution of FSIQ scores between testers (tester 1—mean 74.7, SD 13.2, kurtosis 0.84, skewness 0.08; tester 2—mean 78.6, SD 12.5, kurtosis 0.23, skewness 0.21; P<0.001 (derived from t-test)) and therefore included tester as a covariate in fully adjusted models. Adjusted analyses found smaller, non-significant effect sizes on FSIQ (1.12, 95% CI−0.51 to 2.75; P*=*0.17) and all other intelligence subdomains, as shown in table 3.

In subgroup analyses (table 4), we found no differences in FSIQ between groups among mothers who adhered to 95% of the supplementation (adjusted diff: 1.56, 95% CI −0.44 to 3.56; P=0.13), those with a BMI <18.5 kg/m^2^ (diff: 1.89, 95% CI −1.12 to 4.91; P=0.22), those with haemoglobin (Hb) <11 g/dL during pregnancy (diff: −0.28, 95% CI −3.38 to 3.93; P=0.88) or for children born low birth weight (diff: 2.77, 95% CI −0.92 to 6.45; P=0.14). Table 4 also reports results for subgroups adherence ≤95%, maternal BMI ≥18.5 kg/m^2^, Hb ≥11 g/dL and birth weight ≥2500 g; we found no differences in FSIQ between arms for any of these subgroups. There was a significant interaction between trial allocation arm and child sex (P=0.02) for FSIQ. We therefore conducted a subgroup analysis stratified by sex. Girls in the MMN group had a higher FSIQ than girls in the IFA only group (unadjusted diff: 2.85, 95% CI 0.53 to 5.17; P=0.02).

**Table 4:** Effect of supplements on children’s IQ by adherence, BMI in pregnancy, mothers’ haemoglobin status in pregnancy, birth weight and sex

	Control (n=409) No (%)	Intervention (n=404) No (%)	Difference in means (95% CI)	P value*	Coefficient (95% CI)†	P value
Adherence to supplements						
Children, N	409 (100)	404 (100)	–	–	–	–
Children whose mothers adhered ≥95%, n (%)	277 (67.7)	265 (65.6)	–	–	–	–
Full-scale IQ, mean (SD)	76.5 (13.6)	78.4 (12.2)	1.94 (−0.25 to 4.13)	0.08	1.56 (−0.44 to 3.56)	0.13
Mothers who adhered <95%, n (%)	132 (32.2)	139 (34.4)	–			
Full-scale IQ, mean (SD)	76.1 (14.8)	76.1 (12.3)	−0.06 (−3.30 to 3.19)	0.97	0.40 (−2.47 to 3.27)	0.78
BMI in pregnancy						
Children, N	408 (100)	403 (100)	–	–		
Children whose mothers had BMI <18.5, n (%)	126 (30.9)	120 (29.8)	–	–		0.22
Full-scale IQ, mean (SD)	75.6 (13)	76.3 (13)	0.75 (−2.50 to 4.01)	0.65	1.89 (−1.12 to 4.91)	
Children whose mothers had BMI ≥18.5, n (%)	282 (69.1)	283 (70.2)	–	–	–	
Full-scale IQ, mean (SD)	76.7 (14.5)	78.2 (12)	1.41 (−0.78 to 3.61)	0.21	0.92 (−1.05 to 2.89)	0.36
Haemoglobin (Hb) concentration during pregnancy						
Children, N	409 (100)	404 (100)	–		–	
Children whose mothers had Hb <11 g/dL, n (%)	96 (23.5)	88 (21.8)	–		–	
Full-scale IQ, mean (SD)	78 (15.1)	77.8 (11.7)	−0.12 (−4.08 to 3.84)	0.95	0.28 (−3.38 to 3.93)	0.88
Children whose mothers had Hb ≥11 g/dL, n (%)	313 (76.5)	316 (78.2)	–		–	
Full-scale IQ, mean (SD)	76 (13.6)	77.6 (12.5)	1.67 (−0.37 to 3.71)	0.11	1.26 (−0.56 to 3.07)	0.17
Birth weight						
Children weighed within 72 hours at birth, N	398 (100)	396 (100)	–		–	
Children with birth weight <2500 g	98 (24.6)	71 (17.9)	–		–	
Full-scale IQ, mean (SD)	73.3 (15.1)	75.4 (11.5)	2.12 (−2.10 to 6.34)	0.32	2.77 (−0.92 to 6.45)	0.14
Children with birth weight ≥2500 g	300 (75.4)	325 (82.1)	–		–	
Full-scale IQ, mean (SD)	77.5 (13.5)	78.1 (12.4)	0.55 (−1.48 to 2.49)	0.59	0.51 (−1.35 to 2.37)	0.59
Children’s sex						
Children, N	409	404	–		–	–
Male, n (%)	204 (49.9)	208 (51.5)	–		–	–
Full-scale IQ, mean (SD)	80.4 (12.6)	78.6 (12.1)	−1.75 (−4.15 to 0.64)	0.15	−0.76 (−2.99 to 1.46)	0.50
Female, n (%)	203 (49.6)	196 (48.5)	–	–	–	–
Full-scale IQ, mean (SD)	72.3 (14.2)	76.6 (12.4)	4.25 (1.62 to 6.89)	<0.01‡	2.85 (0.53 to 5.17)	0.02

*Unadjusted, derived from independent-samples t-test.

†Adjusted coefficients derived from linear regression models that included residence (rural vs rural), maternal literacy (cannot read vs can read easily or with difficulty), tester (categorical), HOME score (continuous) and children’s age in days as covariates.

‡Exact P value: 0.0016.

BMI, body mass index; HOME, Early Adolescent Home Observation Tool.

## Discussion

Our follow-up of a double-blind RCT in Nepal found no evidence of overall benefit of antenatal MMN over IFA for children’s intelligence or executive function at 12 years of age. Eight other studies of antenatal MMN supplementation examined cognitive outcomes, and only two found benefits for children’s cognitive function in intention-to-treat analyses. One trial from China by Li et al. found a small increase in mean mental development raw scores using the Bayley Scales of Infant Development at 12 months (1.22, 95% CI 0.32 to 2.12; P=0.02) with MMN supplementation compared with IFA only.^44^ This difference had disappeared by 7–10 years of age.^45^ The second positive study found a 0.11 SD gain (95% CI 0.01 to 0.20, P=0.03) in procedural memory among children whose mothers received antenatal MMN supplementation compared with IFA only.^46^ Three studies found small benefits in subgroup analyses of children born to undernourished or anaemic mothers.^46–48^ The remaining three trials found no benefits of antenatal MMN supplementation compared with placebo or control.^35 49 50^ Online supplementary table 1 describes all eight previous trial follow-up studies and their results. Online supplementary table 2 describes the micronutrients given in antenatal supplementation trials. Except for two trials, all tested the UNIMMAP.^35 49^

10.1136/bmjgh-2017-000527.supp1 Supplementary file 1

With only two trials showing small positive effects and seven, including ours, showing no benefits in mid-childhood or early adolescence, the evidence suggests that antenatal MMN supplementation is unlikely to lead to large, population-level improvements in children’s long-term overall intelligence and executive function. These findings also support evidence from 10 single micronutrient supplementation trials, none of which showed effects on cognitive outcomes. It is possible that any gains in cognitive development seen in infancy, such as those seen in the early study by Li *et al*, are overwhelmed by the subsequent influences of the home and school environments.^6^ It is also possible that follow-up studies of micronutrient supplementation have not measured important domains of cognitive function and missed more subtle effects. For example, only one study measured procedural memory, an ability with distinct neural substrates that appeared to be affected by supplementation.^46^ Future research could explore such effects further.

The positive effect of supplementation on girls’ IQ in this study also warrants further investigation. Earlier analyses of the same trial cohort found no significant sex differences in nutritional status or educational attainment in early life or at 8.5 years.^33 51^ However, in the parent trial, girls exposed to MMN had greater birth weight (diff: 108 g, 95% CI 36 to 179; P=0.003) compared with boys (diff: 44 g, 95% CI 33 to 122; P=0.261).^27^ Sex differences in placentation, fetal growth and response to MMN have been documented.^52 53^ Research on sex-specific DNA methylation patterns in the Gambia suggested that boys and girls followed different developmental trajectories, both with and without supplementation.^54^ Sex-specific responses to MMN supplementation may extend to survival: a meta-analysis of 17 antenatal MMN trials found that neonatal mortality was reduced by 15% in girls and not boys.^55^ In our study, it is possible that female fetuses responded to supplementation differently, and that additional nutrients were directed at weight and the brain. We also cannot rule out the possibility that the effect on girls’ IQ is an artefact of subgroup analyses; it requires exploration in other follow-up studies.

Our study had several strengths. We achieved high rates of follow-up in relation to both the parent trial and the previous follow-up study in 2012. We were also able to assess the balance of allocation on a number of factors that could have confounded the relationship between exposure to micronutrients and intelligence following the initial increase in birth weight found in the parent trial, including the quality of the home environment. Our testers were local people from Dhanusha who were fluent in both of the two local languages, Maithili and Nepali.

Our study also had limitations. We were not able to recruit psychologists to carry out the assessments. We found differences in the mean FSIQ by testers, which may have been due to genuine differences in IQ or bias leading one tester to score some items higher than the other. However, testers were blind to allocation in the parent trial and performed an equal number of tests for children in the MMN and IFA groups. Any tester-induced bias would therefore have affected allocation groups in the same manner, and we included a covariate for tester in adjusted analyses. Two of the instruments—SCARED and HOME—had not been used previously in Nepal. We translated and back-translated tools into Nepali and Maithili and carried out considerable field testing before using them, but some cultural nuances may not have been captured. Mean FSIQ scores were below 100 in both groups, which reflects the fact that the UNIT was standardised with children in the USA. We decided to use standardised scores as these were scaled for age and enabled us to calculate composite scores for all UNIT subscales. Although the standardised scores reflected cultural biases introduced by the US standardisation, we surmised that their effects would be the same across both trial groups.

Why did we find no overall effect of antenatal multiple micronutrient supplementation on FSIQ in early adolescence? Brain development is not restricted to the intrauterine period and is a continuous process, with plasticity enduring into childhood and adulthood. Some have argued that the notion of a ‘critical period’ should not be applied to human brain development.^56^ Rather, one might think of a ‘sensitive period’ with a subsequent broad window of opportunity for continuous development affected by both nutritional and non-nutritional factors, including the environment, stimulation and attachment.^56^ In addition, our analyses may have been underpowered to detect small differences in cognitive outcomes. A 2015 meta-analysis of 18 nutrition interventions including macronutrient or micronutrient supplementation found that these led to a pooled mean effect of 0.086 SD (95% CI 0.034 to 0.137) on children’s cognitive outcomes, a smaller effect than we were powered to detect.^57^

There are several alternative routes to improving children’s cognitive development. Improving maternal nutrition remains key and can be achieved through known nutrition-specific and nutrition-sensitive actions.^2^ *The Lancet*’s 2017 series on child development also recommends interventions that support ‘nurturing care’ for children, defined as a stable environment that is sensitive to their health, nutritional, protection, emotional and learning needs.^58^ Because nurturing care in the early years benefits health, growth and schooling attainment, it is a powerful lever against health and social inequalities.^59^

## Conclusion

In this study, antenatal MMN supplementation did not improve children’s cognitive function, supporting evidence from six previous trials. In order to improve children’s long-term cognitive outcomes, it may therefore be reasonable to prioritise improving women’s nutrition and scaling up access to proven health, nutrition, stimulation and early learning interventions during early childhood.
