# Supplementary material for: Does antenatal micronutrient supplementation improve children’s cognitive function? Evidence from the follow-up of a double-blind randomised controlled trial in Nepal
Source: BMJ Glob Health. 2018 Feb 28;3(1):e000527. doi: 10.1136/bmjgh-2017-000527 (PMC5841533; doi:10.1136/bmjgh-2017-000527)
Supplement: Supplementary file 1 [file bmjgh-2017-000527supp001.pdf]

Supplementary Table 1: Results of follow-up studies of antenatal multiple micronutrient supplementation trials for children's cognitive outcomes

First author (year)	Setting	Age at follow-up Sample size	Measures	Intervention vs. comparison	Results
**McGrath (2006)**	Dar es Salaam, Tanzania	6,12,18 months<br>n = 327	Bayley Scales of Infant Development II	Vitamin A or multiple vitamin only or multiple vitamin + Vitamin A vs. placebo (<28 weeks gestation to postpartum).	No effect of nutrients on cognitive development (-0.7; 95% CI -2.7 to 0.3)
**Tofail (2008)**	Rural Matlab, Bangladesh	7 months<br>n = 2853	One-step means-end Problem-solving Tests (Support and cover test),<br>the motor index of the Bayley Scales of Infant Development, and Wolke's behaviour ratings at seven months of age.	Early food+ Fe30 or Fe 60 or MMN group or usual food + Fe30 or Fe60 or MMN group (14 weeks gestation until delivery).	There were no differences in scores between groups for the problem-solving tests, Bayley Scales and behaviour ratings overall. In a sub-group analysis, children of undernourished mothers performed better in a problem-solving test than those receiving early food supplementation (0.17; 95% CI 0.01 to 0.33; *P*= 0.04).
**Li (2009)**	Rural counties in Western China	3, 6 and 12 months<br>n = 1305	Bayley Scales of Infant Development II	Daily IFA or MMN vs. FA (14 weeks gestation until delivery)	Positive effect of MMN on cognitive development at 12 months in comparison with IFA group (1.22; 95% CI 0.32 to 2.12; *P*=0.02) and folic acid only group (1; 95% CI 0.12 to 1.89; *P*=0.02).
**Christian (2010)**	Sarlahi District, Nepal	7 to 9 years<br>n = 676	UNIT<br>Stroop Numbers Test<br>Backward Digit span<br>Go no-go Inhibitory Control	Daily IFA vs. IFA and zinc vs. MMN vs. Vitamin A alone (pregnancy to 3 months postpartum)	Positive effect of IFA on UNIT score, Stroop test (−0.14; 95% CI, −0.23 to −0.04; P=.005) and backward digit span test scores (0.36; 95% CI, 0.01-0.71; *P*=0.02)<br><br>No difference between control group (IFA) and iron / folic acid / zinc group (0.73; 95% CI −0.95 to 2.42; *P*=0.39) or MMN group (1.00; 95% CI −0.55 to 2.56; *P*=0.20) groups

**Prado (2012)**	Island of Lombok, Indonesia	42 months n = 487	Picture Vocabulary Test<br>Sentence Complexity Scale<br>Block Design Test<br>Visual Search Test<br>Snack Delay Test<br>Windows Test	Daily MMN vs. IFA (Pregnancy to 3 months postpartum)	Positive effect on visual attention/spatial ability in children of undernourished (0.37; 95% CI 0.11 to 0.62; P=0.004) and anemic mothers only (0.24; 95% CI 0.02- to 0.46; *P*= 0.03).
**Hanieh (2013)**	Rural districts of Ha Nam, Vietnam	6 months n = 1258	Bayley Scales of Infant Development III	Twice weekly IFA or twice weekly MMN vs. Daily IFA (<16 weeks gestation to 3 months postpartum)	Positive effect of IFA (in twice weekly group) on cognitive development (MD 1.89; 95% CI 0.23 to 3.56; *P*=0.03)<br>No differences between weekly MMN and daily IFA groups (MD 0.79; 95% CI -0.74 to 2.32; *P*=0.31)
**Li (2015)**	Rural counties in Western China	7-10 years n = 1744	Wechsler Intelligence Scale for Children	Daily IFA or MMN vs. FA (14 weeks gestation until delivery)	No effect on full-scale intelligence quotient (-0.39; CI -1.84 to 1.05) verbal comprehension index (-0.64; 95% CI -2.38 to 1.11), working memory index (0.11; 95% CI -1.26 to 1.48), perceptual reasoning index (-0.43; 95% CI -2.00 to 1.13), processing speed index (-0.34; 95% CI -1.83 to 1.15).
**Prado (2017)**	Island of Lombok, Indonesia	9-12 years n=3068	Information test<br>Speeded picture naming test<br>Block design test<br>Adapted Rey auditory verbal learning test<br>Serial reaction time task<br>Adapted visual search task<br>Digit span forward and backward<br>Stroop numbers<br>NIH Toolbox<br>Dimensional Change Card Sort Test	Daily MMN vs. IFA (Pregnancy to 3 months postpartum)	Children of mothers exposed to antenatal MMN scored 0·11 SD (95% CI 0·01 to 0·20, *P*=0.03) higher in procedural memory compared to those exposed to IFA only.<br>Children of anaemic mothers exposed to antenatal MMN scored 0·18 SD (0·06–0·31, *P* =.005) higher in general intellectual ability compared to those exposed to IFA only.

Supplementary Table 2: Summary of micronutrients given in antenatal supplementation trials

Study	Intervention	Vitamin A (µg)	Vitamin B1 (mg)	Vitamin B2 (mg)	Vitamin B3 (mg)	Vitamin B6 (mg)	Vitamin B12 (µg)	Folic acid (mg)	Vitamin C (mg)	Vitamin D (IU)	Vitamin E (mg)	Iron (mg)	Zinc (mg)	Copper (mg)	Iodine (µg)	Selenium (µg)	Choline (mg)	Potassium (µg)	Magnesium (mg)
**McGrath 2006**	Multivitamin	-	20	20	100	25	50	0.8	500	-	30	-	-	-	-	-	-	-	-
	Multivitamin + Vitamin A	6000	20	20	100	25	50	0.8	500	-	30	-	-	-	-	-	-	-	-
	Vitamin A	6000	-	-	-	-	-	-	-	-	-	-	-	-	-	-	-	-	-
**Tofail**	Food MMN	800	1.4	1.4	18	1.9	2.6	0.4	70	200	10	30	15	2	150	65	-	-	-

**2008**	Food Fe60	-	-	-	-	-	-	0.4	-	-	-	60	-	-	-	-	-	-	-
	Food Fe30	-	-	-	-	-	-	0.4	-	-	-	30	-	-	-	-	-	-	-
**Li 2009**	MMN	800	1.4	1.4	18	1.9	2.6	0.4	70	200	10	30	15	2	150	65	-	-	-
	IFA	-	-	-	-	-	-	0.4	-	-	-	60	-	-	-	-	-	-	-
	FA	-	-	-	-	-	-	0.4	-	-	-	-	-	-	-	-	-	-	-
**Christian 2010**	MMN	1000	1.6	1.8	20	2.2	2.6	0.4	100	400	10	60	30	2	-	-	-	65	100
	IFA + zinc	-	-	-	-	-	-	0.4	-	-	-	60	30	-	-	-	-	-	-
	IFA	-	-	-	-	-	-	0.4	-	-	-	60	-	-	-	-	-	-	-
	FA	-	-	-	-	-	-	0.4	-	-	-	-	-	-	-	-	-	-	-
**Prado 2012**	MMN	800	1.4	1.4	18	1.9	2.6	0.4	70	200	10	30	15	2	150	65	-	-	-
	IFA	-	-	-	-	-	-	0.4	-	-	-	30	-	-	-	-	-	-	-

Study	Intervention	Vitamin A (µg)	Vitamin B1 (mg)	Vitamin B2 (mg)	Vitamin B3 (mg)	Vitamin B6 (mg)	Vitamin B12 (µg)	Folic acid (mg)	Vitamin C (mg)	Vitamin D (IU)	Vitamin E (mg)	Iron (mg)	Zinc (mg)	Copper (mg)	Iodine (µg)	Selenium (µg)	Choline (mg)	Potassium (µg)	Magnesium (mg)
**Hanieh 2013**	MMN	800	1.4	1.4	18	1.9	2.6	0.4	70	200	10	30	15	2	150	65	-	-	-
	Twice weekly IFA	-	-	-	-	-	-	1.5	-	-	-	60	-	-	-	-	-	-	-
	Daily IFA	-	-	-	-	-	-	0.4	-	-	-	60	-	-	-	-	-	-	-
**Li 2015**	MMN	800	1.4	1.4	18	1.9	2.6	0.4	70	200	10	30	15	2	150	65	-	-	-
	IFA	-	-	-	-	-	-	0.4	-	-	-	60	-	-	-	-	-	-	-

	FA	-	-	-	-	-	-	0.4	-	-	-	-	-	-	-	-	-	-	-
**Prado 2017**	MMN	800	1.4	1.4	18	1.9	2.6	0.4	70	200	10	30	15	2	150	65	-	-	-
	IFA	-	-	-	-	-	-	0.4	-	-	-	30	-	-	-	-	-	-	-

Notes:

Christian 2010 and McGrath 2005 did not use United Nations International Multiple Micronutrient Preparation (UNIMMAP).

McGrath 2005 - Vitamin A dose provided as 30 mg beta-carotene (4500 µg RAE) and 5000 IU (1500 µg RAE) preformed vitamin A

Tofail 2008 - Food packet contained roasted rice powder (80 g), roasted lentil powder (40 g), molasses (20 g), and soy oil (12 mL)

Christian 2010 - IFA for children provided in combined value - 12.5mg
